# Nitric-acid

**Published:** 1896-10

**Authors:** 


### HOMŒOPATHIC PHYSICIAN,

A MONTHLY JOURNAL OF

HOMŒOPATHIC MATERIA MEDICA AND CLINICAL MEDICINE.

"If our school ever gives up the strict inductive method of Hahnemann, we are lost, and deserve only to be mentioned as a caricature in the history of medicine."—CONSTANTINE HERING.

Vol. XVI. OCTOBER, 1896. No. 10.

### EDITORIAL.

NITRIC-ACID.—This remedy is the great antidote to Mercury in large doses. It has many resemblances to Mercury, and therefore should naturally follow Mercury in a consideration of symptomatology.

Taking the symptoms individually, the Nitric-acid patient has fear of death, yet he is tired of life. He is disinclined to talk, thus resembling Phosphoric-acid. He has weakness of memory, with no inclination to mental exertion. Calcarea-carbonica has utter impossibility of mental exertion.

This symptom of mental effort suggests a number of comparisons.

Mental exertion, accompanied by torn feeling in the throat. Causticum.

Mental exertion causes sense of helplessness from brain weakness. Can't attend to anything requiring thought. Gelsemium.

Can't fix his mind on his studies. Iris-v.

Mental exertion causes pain in occipital region. Picric-acid.

Mental exertion aggravates headache and produces sleep. Spigelia.

Mental exertion aggravates headache. Colchicum, Glonoinum, Natrum-muriaticum, and Nux-vomica.

Mental exertion aggravates pressing, boring headache. Nux-vomica.

Mental exertion to excess produces irritable stomach. Nux-moschata.

Mental exertion aggravates mental symptoms. Nux-vomica.

Mental exertion aggravates all ailments. Nux-vomica.

Mental exertion in excess causes bad effects, as in bookkeepers, etc. Anacardium and Sepia.

Mental exertion causes fullness in head, intense headache, throbbing in the brain and pain in left temple. Psorinum.

Aggravation from mental exertion. Aurum-metallicum, Carbo-vegetabilis, Colchicum, Magnesia-carbonica, Nux-vomica.

Mental exertion aggravates hemorrhoids. Causticum.

Mental exertion causes bruised soreness all over the head. China.

Mental exertion causes pressing, boring headache. Colchicum.

The least mental exertion causes headache. Aurum-metallicum.

Mental exertion aggravates the headache that follows a suppressed coryza. China.

Mental exertion causes pressing pains in the head. Argentum-nitricum.

Mental exertion causes him to become stupid with headache in forehead. Petroleum.

Mental exertion causes pressure in occiput and deep in cerebellum. Colchicum.

Mental exertion moderates or improves many ailments. Ferrum.

Mental exertion causes aggravation of pressing pain on top of head and in left frontal bone, with running together of letters when reading. Argentum-nitricum.

Mental exertion or fixing thoughts even for a moment on anything is impossible, causing headache and darkness before the eyes. Argentum-nitricum.

Nitric-acid has vertigo on rising from the bed. This is similar to Phosphorus.

Nitric-acid has sensitiveness of the head to the slightest pressure, even the pressure of the hat.

Carbo-vegetabilis also has sensitiveness of the head to the pressure of the hat. Carbo-animalis has the same symptom. Glonoine has the same symptom. The patient can't bear the hat on the head at all.

Calcarea-phosphorica has aggravation of fullness and sense of pressure in the head from the pressure of the hat.

Nitric-acid has sensation in the bones of the skull as if constricted with a tape. Mercurius has the same sensation in the bones of the forehead. Indigo has a similar symptom.

The editor has collected the following additional comparisons :

Sensation of a band in frontal region. Merc-jodat-ruber.

Sensation of a band around the head above the ears. Scalp sore. Gelsemium.

Sensation of a band around the head, with inflammation of the brain ; burning and pulsation in the forehead ; worse at night and better after riding. Mercurius.

Constriction as of a band around the head, from overheated rooms ; congestion to the head ; coryza and nausea. Carbo-veg.

Sensation of a band around the head with headache ; worse from stooping. Spigelia.

Sensation of a tense band around the back of the head, reaching from the nape of the neck to the ears. Anacardium.

Headache with a sensation of a band of hot iron bound around the head. Aconite.

Headache better from tying a band around the head. Hepar.

Sensation of a band around the forehead. Baptisia.

Desire for a band around the head. Apis.

Other parts of the body have this sensation of being encircled with a band.

Sensation as if the legs were bandaged. Benzoic-acid.

Sensation of a band around the chest, with an irresistible desire to cough. Lobelia.

Sensation of band around different parts. Sabadilla and Sulphur.

Sensation of a band around the arms, and the blood rushing into the hands in the evening. Nux-moschata.

Constriction as if from a tight band around the abdomen. Argentum-nitricum.

Sensation of a tight band around lower abdomen, or as if laced tightly. Ignatia.

Sensation as if knees were firmly bandaged when sitting. Aurum-metallicum.

Sensation as if the thighs were bandaged. Aconite.

Sensation as if the legs were tightly bandaged. Benzoic-acid.

